# Evidence for Age-Equivalent and Task-Dissociative Metacognition in the Memory Domain

**DOI:** 10.3389/fpsyg.2021.630143

**Published:** 2021-02-05

**Authors:** Alexandria C. Zakrzewski, Edie C. Sanders, Jane M. Berry

**Affiliations:** ^1^Department of Psychological Sciences, Kansas State University, Manhattan, KS, United States; ^2^Department of Psychology, Florida State University, Tallahassee, FL, United States; ^3^Department of Psychology, University of Richmond, Richmond, VA, United States

**Keywords:** aging, associative recognition, metacognition, metacognitive efficiency, associative deficit hypothesis

## Abstract

Research suggests that metacognitive monitoring ability does not decline with age. For example, judgments-of-learning (JOL) accuracy is roughly equivalent between younger and older adults. But few studies have asked whether younger and older adults’ metacognitive ability varies across different types of memory processes (e.g., for items vs. pairs). The current study tested the relationship between memory and post-decision confidence ratings at the trial level on item (individual words) and associative (word pairs) memory recognition tests. As predicted, younger and older adults had similar *metacognitive efficiency*, when using meta-*d’/d’*, a measure derived from Signal Detection Theory, despite a significant age effect favoring younger adults on memory performance. This result is consistent with previous work showing age-equivalent metacognitive efficiency in the memory domain. We also found that metacognitive efficiency was higher for associative memory than for item memory across age groups, even though associative and item recognition memory (*d’*) were statistically equivalent. Higher accuracy on post-test decision confidence ratings for associative recognition relative to item recognition on resolution accuracy itself (meta-*d’*) and when corrected for performance differences (meta-*d’/d’*) are novel findings. Implications for associative metacognition are discussed.

## Introduction

Metacognition or “thoughts about one’s own thoughts and cognition” (Flavell, 1979) includes monitoring judgments about how well one learns, solves problems, reasons, and retrieves memories. Metacognition is important for regulating behavior (Nelson and Narens, 1990). The accuracy of our metacognitive judgments is critical because these judgments impact decision-making. Ideally, our confidence level matches our cognitive ability. Metacognitive accuracy, or how well subjective judgments about performance (e.g., confidence) match performance accuracy, is often assessed at the trial level of a task. When one makes high confidence ratings for correct responses and low confidence ratings for incorrect responses, s/he has good metacognitive accuracy. Poor metacognitive accuracy is indicated by a mismatch between confidence level and performance accuracy.

Metacognitive accuracy is especially important as we age. Metacognitive accuracy in cognitive, physical, and health domains could translate to pertinent help-seeking behaviors. For example, if an older adult is experiencing memory problems but fails to recognize these problems due to poor metacognitive ability, they may fail to adhere to a medication or diet, or other behaviors crucial to good health outcomes. This failure can have negative effects on one’s quality of life and can exacerbate the progression of memory problems. Therefore, understanding metacognitive aging could provide crucial insight into possible interventions.

A robust body of research shows that metacognitive accuracy remains relatively intact in late life (e.g., Connor et al., 1997; Dunlosky and Hertzog, 2000; Hertzog et al., 2002, 2021; Sanders and Berry, 2020); this holds across different types of stimuli, memory tests, and metacognitive assessments. For example, using a 5AFC recognition task for studied category exemplars, Hertzog et al. (2021) found that resolution of retrospective confidence judgments (RCJs) was the same across age groups, despite negative age differences in recognition memory. Likewise, Sanders and Berry (2020) found comparable monitoring resolution using emotionally valenced words and judgments-of-learning (JOLs) on a 2AFC recognition task, despite negative age differences in recognition accuracy, and Berry et al. (2013) reported age-equivalent metacognitive (“postdiction”) accuracy on item and associative tasks. These effects generalize to studies where memory is equated between younger and older adults on an associative memory task, using RCJ resolution (Hines et al., 2009). Together, these studies suggest that older adults (OAs) may be aware of age-related declines in cognitive abilities. However, in other studies, OAs produce less accurate RCJs than younger adults (YAs) while exhibiting age-related deficits in recognition memory (Wong et al., 2012) and cued recall (Kelley and Sahakyan, 2003). Thus, the conditions vary by which metacognitive accuracy is spared or impaired in older adults.

Metacognitive accuracy might also vary across different cognitive domains (e.g., perception, memory) and for different types of retrieval processes within a domain such as memory (e.g., recall, recognition). The question of whether metacognition generalizes across domains is receiving increased research attention (e.g., Fleming et al., 2014; Fitzgerald et al., 2017; Morales et al., 2018). Palmer et al. (2014) found that metacognitive accuracy decreased with age in a perception task but not in a memory task, suggesting a domain-specific age effect despite domain-general evidence (e.g., Mazancieux et al., 2020). To our knowledge, the effects of age on metacognitive accuracy between different tasks in the memory domain has not been examined.

Memory research shows that individuals are generally more accurate at recognizing items (e.g., individual words) than associates (e.g., word pairs). This effect is moderated by age. Specifically, the difference between memory for items vs. pairs of stimuli increases with age (e.g., Naveh-Benjamin et al., 2009; Badham and Maylor, 2011; Fox et al., 2016), as predicted by the associative deficit hypothesis (ADH; Naveh-Benjamin, 2000). Given the superiority of item over associative recognition accuracy in most tests of the ADH, one might expect metacognitive accuracy to reflect this dissociation. However, age differences in metacognition do not always reflect age differences in memory (e.g., Connor et al., 1997; Hertzog et al., 2002; McGillivray and Castel, 2011). Indeed, Hertzog et al. (2010a) demonstrated that resolution accuracy for feelings-of-knowing varied by quality of encoding at study, and this held across age groups.

Metacognitive accuracy might track the age-related associative deficit, supporting work that points to the possible role of metacognition for OAs’ associative memory failure (e.g., Naveh-Benjamin et al., 2005, 2007; Bender and Raz, 2012). That is, item metacognition might be superior to associative metacognition. Alternatively, metacognitive accuracy could be better for associates than items if the quality of encoding paired-associates enhanced metacognitive accuracy (Hertzog et al., 2010a). Paired associates elicit encoding strategies such as sentence generation and interactive imagery mediators that serve to help bind words together during study (see Dunlosky and Hertzog, 1998). Mediators can serve as cues for confidence (Koriat, 1997) and, if used effectively, could contribute to superior metacognitive efficiency in associative recognition. For example, Hertzog et al. (2010b) showed that generation of imagery and sentence mediators during a paired-associate task influenced memory, JOLs, and JOL resolution (measured by gamma coefficients) in a large cross-sectional sample of adults. Additionally, in a 5AFC item recognition test, Hertzog et al. (2021) found that orienting participants to encode distinct rather than shared features of category exemplars enhanced item recognition accuracy and confidence judgment accuracy. Distinctive encoding also reduced high-confidence false alarms (Shing et al., 2009; Fandakova et al., 2013). Given the benefits of encoding strategies on metacognition, metacognitive accuracy might be better on an associative test than an item test, when imagery, sentence generation, and other mediators created during study could be less influential.

The present study investigated the impact of age on metacognition for item (individual words) and associative (word pairs) recognition tests. We used a measure of metacognition derived from Signal Detection Theory: Meta-*d’/d’* or metacognitive efficiency (Maniscalco and Lau, 2012). Meta-*d’/d’* has been used to assess metacognitive accuracy in word recognition (Baird et al., 2013; McCurdy et al., 2013; Palmer et al., 2014; Carpenter et al., 2019), pattern recognition (Lee et al., 2018), and semantic and word-number sequency memory tasks (Mazancieux et al., 2020). We asked YAs and OAs to study word pairs. At test, participants indicated whether or not they recognized the individual words (items) or word pairs (associates) and made trial-level confidence ratings about the accuracy of their responses. We predicted equivalent metacognitive efficiency for YAs and OAs given the preponderant evidence that metacognitive ability is spared in OAs. We also tested task type differences in metacognitive efficiency for associative recognition and item recognition.

## Materials and Methods

### Design and Participants

The experiment utilized a mixed design with age group (YA, OA) as a between-subjects factor and test type (item, associative) as a within-subjects factor. Twenty-eight YAs (15 female) aged 18–21 (*M* = 19.25, *SD* = 1.04) were recruited from introductory psychology classes and received course credit for participation. Twenty-nine OAs (19 female) aged 64–85 (*M* = 73.22, *SD* = 5.35) were recruited from the surrounding community through newspaper ads and a participant database. They received $20 for participation. The entire experiment lasted approximately 1 h and 20 min. Four YAs and 5 OAs were dropped because their discriminability (*d’*) on the item or associative test was 0.15 or below. This exclusion criterion was adopted because it is difficult to compute stable metacognitive efficiency when performance accuracy is low. The final dataset included 49 participants (24 YAs, 25 OAs). Table 1 reports demographic data and standardized scores on processing speed and vocabulary measures.

**TABLE 1:** Means, standard deviations, and effect sizes for demographic comparisons between age groups.

**Variable**	**Younger adults**	**Older adults**	**Age effect size**
	**(*N* = 24)**	**(*N* = 25)**	**(Cohen’s *d*)**
% Female	50.0	68.0	–
Age	19.38 (1.06)	73.30 (4.86)	–
Years of education	13.13 (0.95)	15.80 (1.98)	1.71^∗∗∗^
Self-rated health	8.33 (1.55)	8.76 (1.13)	0.32
Self-rated vision	8.54 (1.69)	8.74 (1.23)	0.13
Self-rated hearing	8.71 (1.23)	8.04 (2.09)	–0.39
Speed of processing^a^	69.08 (10.72)	49.52 (9.08)	–1.97^∗∗∗^
Vocabulary^b^	25.58 (2.86)	29.64 (2.56)	1.50^∗∗∗^

Scales for self-rated health, vision, and hearing ranged from 0 (poor) to 10 (excellent). ^*a*^Digit Symbol Substitution Test (DSST; Wechsler, 1981). ^*b*^Synonyms Test (Ekstrom et al., 1976). ***p < 0.001.

### Materials

The stimuli were English words generated using the Medical Research Council Psycholinguistic Database (Wilson, 1988). Standard nouns with 4–10 letters (*M* = 5.68; *SD* = 1.43) and one to two syllables (*M* = 1.55; *SD* = 0.50) were retrieved by the database and had familiarity, concreteness, and imageability ratings of 200–700 each^1^. We randomly sorted and paired words retrieved from the database, constructing four sets of 30 pairs of words as well as four sets of 20 new individual words/lures for the item recognition tests. The Edinburgh Associative Thesaurus (EAT; Kiss et al., 1973) was used to calculate the association strength between words in each word pair. Any pairs with a distance of three “nodes” or less were excluded from the final stimulus set.

### Procedure

Participants were brought into a quiet testing room and seated at a computer. The study was introduced, informed consent obtained, and a demographic questionnaire administered.

**FIGURE 1:**
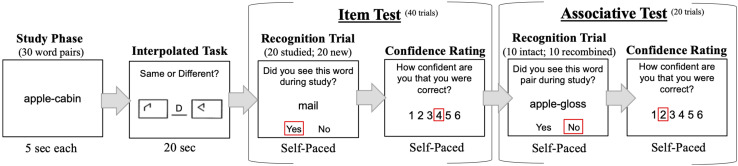
Example of task procedure. For each block, participants studied 30 word-pairs and completed an interpolated task to prevent rehearsal, followed by 40 item and 20 associative test trials. Participants made a confidence rating after each test trial. The entire experiment consisted of four blocks. The order of item and associative tests was counterbalanced across participants.

Recognition tasks were programmed and run on E-Prime version 2.0. The task procedure for a single experimental block is illustrated in Figure 1.

Participants completed four experimental blocks. Each block comprised a study phase, interpolated task, item recognition test, and associative recognition test. The study phase, item test, and associative test were modeled after the procedure of Naveh-Benjamin (2000) and other work testing the ADH (e.g., Naveh-Benjamin et al., 2007, 2009; Badham and Maylor, 2011; Ratcliff and McKoon, 2015; Bartsch et al., 2019). Between block 2 and 3, participants were offered a break to rest. During study, 30 word-pairs were presented sequentially for 5 s each. Participants were instructed to study individual words and their pairings. A new study list of 30 word-pairs was presented in each of the four blocks. Words were never re-used across blocks or from the practice session. After study, participants completed the Salthouse (1991) 20 s pattern comparison task to limit rehearsal of studied words. Participants then began either the item or associative recognition test. The order of item and associative recognition tests was counterbalanced across participants. If a participant was assigned to complete item before associative tests, the same order was implemented in all blocks. During the item test, 40 words (20 studied; 20 new) were presented sequentially in random order. During each recognition trial, a studied or new word appeared beneath the question “Did you see this word during study?.” Participants were instructed to respond “Y” or “N” on the keyboard for “yes” or “no.” During the associative test, 20 pairs were presented sequentially in random order. Of these 20 pairs, 10 were intact from the study list and 10 were recombined by combining words that had appeared in previously studied pairs into new pair configurations. During each recognition trial, an intact, or recombined word-pair was presented beneath the question “Did you see this pair during study?” Participants were instructed to respond “Y” or “N.”

Before recognition tests, participants were instructed to make a confidence rating using labeled keys on the keyboard. Instructions on the screen stated: “After each response, make a confidence rating (1 through 6) about the accuracy of your response; 1 = Low Confidence 6 = High Confidence; Please use the entire scale to make a relative confidence judgment.” During item and associative tests, immediately following each “Y” or “N” response, participants were asked “How confident are you that you were correct?” Participants had unlimited time to make each recognition response and confidence rating. After each confidence rating was made, a new recognition trial began.

Before the experimental blocks began, participants completed a short practice block, which was a shorter version of an experimental block. The procedure was the same, except for the number of word pairs studied and tested: Study: 6 word pairs; Item Test: 4 words (2 studied, 2 new); Associative Test: 4 word pairs (2 intact, 2 recombined).

After block 4, participants completed a post-test questionnaire and measures of processing speed and vocabulary knowledge in order to characterize our sample’s age differences in cognition and comparability to other cognitive aging research (see Table 1). Older adults typically show larger vocabulary knowledge (Verhaeghen, 2003) but slower processing speed (Hoyer et al., 2004). Participants were debriefed and compensated for their participation.

## Results

### Statistical Analyses

Metacognitive efficiency was characterized with a measure of metacognition (meta-*d’*) that takes into account an individual’s level of performance sensitivity (*d’*): meta-*d’/d’*, calculated using MATLAB code available at http://www.columbia.edu/~bsm2105/type2sdt/ (Maniscalco and Lau, 2012). Meta-*d’* reflects the degree to which a participant’s confidence ratings can discriminate between correct and incorrect responses (Barrett et al., 2013; Fleming and Lau, 2014). Because meta-*d’* uses the same scale as *d’*, a measure of relative metacognitive sensitivity (metacognitive efficiency) can be calculated by dividing meta-*d’* by *d’*, taking into account the potentially confounding influences of task performance (*d’*). A meta-*d’/d’* value of 1 indicates that metacognitive sensitivity (meta-*d’*) matches task sensitivity (*d’*). Meta-*d’/d’* greater than or less than 1 indicates metacognition exceeds or fails to reach task performance, respectively. For example, a meta-*d’/d’* value of 0.7 would indicate 70% metacognitive efficiency (Fleming and Lau, 2014).

We used JASP (JASP Team, 2020) to conduct 2 × 2 mixed design analyses with age (YA, OA) as the between-participants factor and task type (item, associative) as the within-participants factor. Dependent variables were meta-*d’/d’*, meta-*d’*, and *d’*. For *post hoc* tests, the Holm-Bonferroni method was used for independent and dependent samples *t-*tests to maintain an alpha level of 0.05.

To supplement the two-way ANOVA tests of our hypothesis of age-equivalent metacognitive efficiency, we calculated Bayes factor (BF) (Faulkenberry, 2019)^2^.

We chose our initial sample size based on similar studies in the metacognitive aging literature for a 2 × 2 mixed ANOVA (e.g., McGillivray and Castel, 2011). We conducted power analyses at the conclusion of our study using G^∗^Power (Faul et al., 2007), setting α to 0.05, power to 0.95, and ES to 0.33 (η*${}_{\text{p}}^{2}$* = 0.10), which yielded *N* = 32, lower than our initial *N* = 49. These results derive from the *a priori* option, which we should have chosen prior to collecting data. We also ran the *post hoc* power analysis using α = 0.05, ES = 0.71 (performance η*${}_{\text{p}}^{2}$* = 0.052), and *N* = 49, using the obtained ANOVA results for *d’*. The *post hoc* analysis yielded power = 1.0. These analyses suggest that our tests were sufficiently powered.

### Metacognitive Efficiency (Meta-*d’/d’*)

The effect of age on meta-*d’/d’* was non-significant, *F*(1, 47) = 0.748, *p* = 0.392, η*_p_*^2^ = 0.016, supporting our prediction of age-invariant metacognitive efficiency for YAs (*M* = *0.96*, *SD* = 0.08) and OAs (*M* = 1.05, *SD* = 0.08). Moreover, both age groups were highly accurate in metacognitive skill. Single-sample *t*-tests comparing mean metacognitive efficiency scores to a value of 1.00 (i.e., optimal metacognitive efficiency) revealed that both YAs (*M* = 0.96, *SD* = 0.36) and OAs (*M* = 1.05, *SD* = 0.37) were close to optimal in metacognitive efficiency: YA *t*(24) = −0.560, *p* = 0.581, and OA *t*(25) = 0.663, *p* = 0.514.

Our Bayes factor analysis yielded BF_01_ = 4.75, indicating that the observed data favored the null hypothesis of age equivalence for metacognitive accuracy over the alternative by about 5–1, M_dif_ = -0.09, *t*(47) = -0.865, *p* = 0.392. The posterior probability for the null is 0.83 and for the alternative is 0.17. These results lend further support for our prediction of age-equivalent metacognitive efficiency.

**FIGURE 2:**
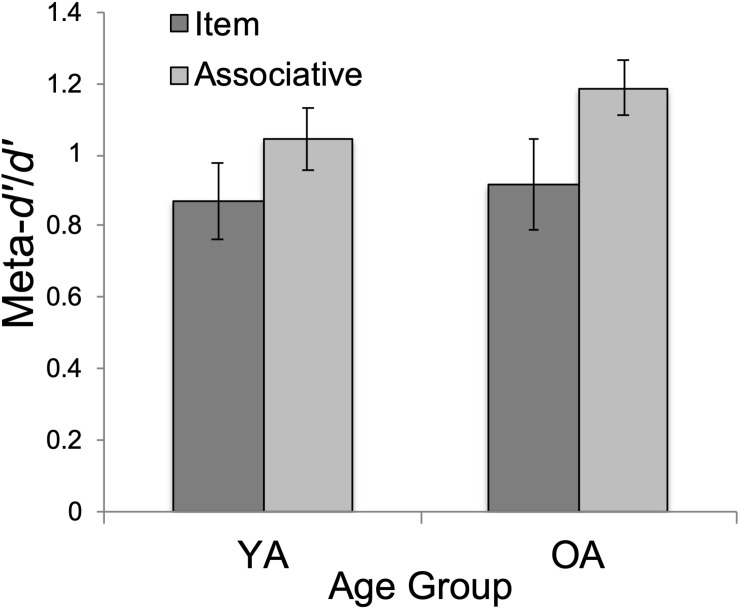
Mean metacognitive efficiency (meta-*d’/d’*) by age group (YA = younger adults; OA = older adults) and test type (item/associative). Errors bars show standard error.

There was a significant main effect of test type, *F*(1, 47) = 5.43, *p* < 0.05, η*_p_*^2^ = 0.104. Associative meta-*d’*/*d’* (*M* = 1.12, *SD* = 0.58) was significantly higher than item meta-*d’*/*d’* (*M* = 0.90, *SD* = 0.41), *t*(47) = 2.33, *p_bonf_* = 0.024, Cohen’s *d* = 0.333 (see Figure 2).

The age by task type interaction effect was non-significant (*F* < 1).

#### Metacognitive Sensitivity (Meta-*d’*)

We also examined age and task type effects on metacognitive sensitivity (meta*-d’*) unadjusted for performance sensitivity (*d’*). Meta-*d’* reflects how well one can distinguish between one’s correct and incorrect judgments (Fleming and Lau, 2014).

The effect of age on meta-*d’* was non-significant, *F*(1, 47) = 2.05, *p* = 0.158, η*${}_{\text{p}}^{2}$* = 0.042, indicating age-invariant metacognitive sensitivity for YAs (*M* = 1.57, *SD* = 0.88) and OAs (*M* = 1.29, *SD* = 0.75). A Bayes factor of BF_01_ = 2.46 indicated that the observed data favored the null hypothesis of age equivalence for resolution accuracy over the alternative by about 2.5–1. The posterior probability for the null is 0.71 and for the alternative is 0.29.

There was a significant main effect of test type, *F*(1, 47) = 6.51, *p* < 0.05, η*_p_*^2^ = 0.122. Associative meta-*d’* (*M* = 1.59, *SD* = 0.97) was significantly higher than item meta-*d’* (*M* = 1.27, *SD* = 0.62), *t*(47) = 2.55, *p*_bonf_ = 0.014, Cohen’s *d* = 0.364.

The age by task type interaction effect was non-significant (*F* < 1).

We also analyzed age group and test type effects on Goodman-Kruskal gamma coefficients because gamma is a widely used estimate of resolution accuracy in the metacognitive aging literature. None of the ANOVA effects were significant (see Supplementary Materials). Thus, using signal-detection measures to assess metacognitive sensitivity and metacognitive efficiency revealed task-type effects that were not revealed by the more commonly used measure of metacognitive accuracy. Note that both measures yielded consistent age results, that is, equivalent resolution accuracy between YA and OA^3^.

### Recognition Memory Performance (*d’*)

**FIGURE 3:**
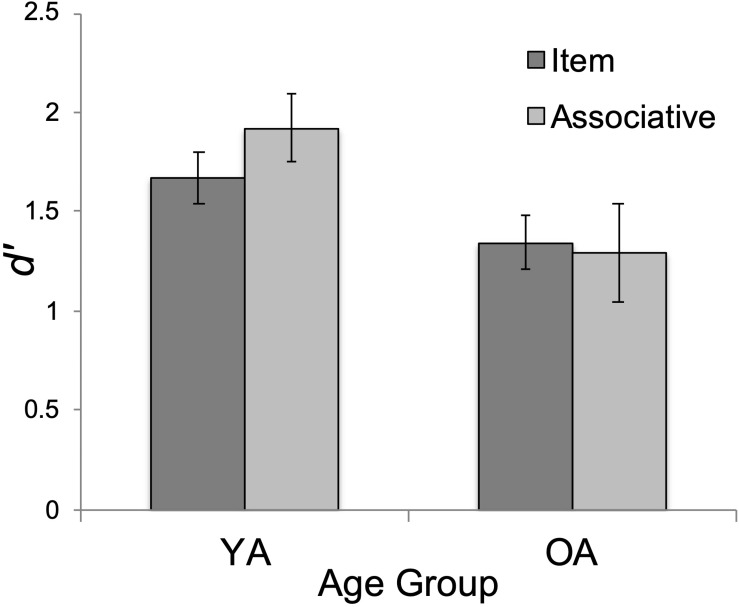
Mean performance (*d’*) by age group (YA = younger adults; OA = older adults) and test type (item/associative). Errors bars show standard error.

As expected, the main effect of age on *d’* was significant, *F*(1, 47) = 4.19, *p* = 0.046, η*_p_*^2^ = 0.082. YAs’ overall performance (*M* = 1.80, *SD* = 0.97) exceeded that of OAs (*M* = 1.32, *SD* = 0.77), *t*(47) = 2.05, *p*_bonf_ = 0.046, Cohen’s *d* = 0.292 (see Figure 3). The main effect of test type was not significant, *F* < 2. Surprisingly, the interaction between test type and age group was not significant (*F* < 3, *p* = 0.116, η*_p_*^2^ = 0.052).

We also conducted exploratory *t*-tests between age groups on the item and associative *d’* scores. Interestingly, YA’s associative *d’* (*M* = 1.92, *SD* = 1.20) was significantly greater than OA’s (*M* = 1.30, *SD* = 0.87), *t*(47) = 2.09, *p* = 0.04, Cohen’s *d* = 0.60 whereas age effects were non-significant on the item task, *t*(47) = 1.72, *p* = 0.09, Cohen’s *d* = 0.49 (YA: *M* = 1.67, *SD* = 0.65; OA: *M* = 1.35, *SD* = 0.67). The pattern of these mean differences is in the direction predicted by the ADH. However, because the interaction effect was non-significant, we report these mean comparisons with caution.

## Discussion

### Metacognition and Age

First, we tested age differences in metacognitive efficiency. Our prediction for equivalent metacognitive efficiency (meta-*d’/d’*) between YAs and OAs was supported. This finding supports the growing consensus that OAs retain their ability to monitor memory performance accuracy, despite age-normative declines in memory performance (Shaw and Craik, 1989; Connor et al., 1997; Dunlosky and Hertzog, 2000; Hertzog et al., 2002, 2021; Sanders and Berry, 2020). As in Palmer et al. (2014)’s study, we observed age-equivalent metacognitive efficiency for memory (Figure 2), despite worse recognition memory for OAs, overall (Figure 3).

### Metacognition and Task Type

Second, we tested memory type (item, associative) differences in metacognitive efficiency. Metacognitive efficiency was greater for associative recognition than for item recognition, despite equivalent task type performance (Figure 3). This fits well with evidence from memory research on the benefits of elaborative encoding strategies (e.g., interactive imagery, sentence generation) to create an association or bind studied items, such as word pairs, during associative tasks (e.g., Beuhring and Kee, 1987; Hertzog and Dunlosky, 2004; Naveh-Benjamin et al., 2007; Hertzog et al., 2010a; Hawco et al., 2013). Mediators and other binding cues during encoding could support more accurate confidence ratings for pairs than individual items.

### Memory Performance

Recognition accuracy was worse for OAs than YAs, as expected. Our results failed to confirm the ADH because the interaction effect between age and test type was non-significant, contrary to previous findings (e.g., Naveh-Benjamin, 2000; Naveh-Benjamin et al., 2009; Badham and Maylor, 2011; Berry et al., 2013; Fox et al., 2016). However, *post hoc* tests of age differences on *d’* showed that our OA scored more poorly than YAs on the associative task. We conducted a follow-up study to test the effects of confidence ratings on performance^4^.

### Implications

The role of age differences in metacognition is interesting because they do not necessarily track age differences in memory, as demonstrated in our study and others. For example, McGillivray and Castel (2011) asked OAs and YAs to make bets about whether they would remember each word later in a free recall task. Initially, both YAs and OAs bet on more items than they could recall, overestimating their memory. However, as the task progressed, both age groups calibrated their betting behavior based on experience and feedback. Despite OAs’ recall performance remaining significantly worse, OAs and YAs ended the task with equivalent “betting accuracy.” In fact, OAs were marginally better calibrated than YAs. Additionally, in paired-associate recall tasks, OAs made relatively accurate JOLs despite age-related memory deficits on the memory task (Hertzog et al., 2002; see also Connor et al., 1997). Like these studies, our present results demonstrated that metacognitive ability remains intact despite an age-related memory impairment (also, see Lovelace and Marsh, 1985; Shaw and Craik, 1989; Connor et al., 1997; Dunlosky and Hertzog, 1997).

Furthermore, ours is the first study to show that associative metacognition is superior to item metacognition and this difference existed across age groups, even when associative and item memory did not differ. It could be the case that enhanced associative metacognition is driven by different mechanisms for YAs and OAs, or individual differences within age groups. For example, artificial neural network modeling revealed metacognitive differences between and within age groups (Zakrzewski et al., 2019). Thus, while metacognition might look the same for YAs and OAs, it is not necessarily the case that YAs and OAs used the same strategies or relied on the same metacognitive cues.

### Limitations

One might argue that, because OAs often fail to use elaborative strategies, metacognition would reflect this failure, showing poorer metacognitive accuracy on the associative task. Because our study failed to reveal a significant age by task type interaction effect, as in most studies on the ADH (e.g., Naveh-Benjamin, 2000), it is difficult to argue that OAs’ accurate confidence ratings reflect awareness of this deficit. Furthermore, it is important to test how metacognitive accuracy might vary when performance does not, since metacognitive differences can be confounded by memory differences (see Perfect and Stollery, 1993). Hertzog et al. (2021) found that when performance was matched, age differences in high confidence errors disappeared.

Future work should explore whether associative metacognition is superior to item metacognition in other tasks. For example, Kelley and Sahakyan (2003) found OAs made less accurate confidence ratings than YAs in a cued recall task. Meta-*d’/d’* would have to be calculated differently for cued or free recall tasks due to the restraints of signal detection modeling. However, the present framework could be extended to other tasks, such as those involving non-verbal stimuli including pictures or numbers (e.g., Ratcliff and McKoon, 2015; Lee et al., 2018; Mazancieux et al., 2020). Geurten and Lemaire (2019) found similar metacognitive ability across age groups completing arithmetic tasks. Results found here might vary in different domains, as Palmer et al. (2014) showed, as well as tasks that utilize different retrieval formats, such as the 5AFC employed by Hertzog et al. (2021). Hertzog and Dunlosky (2011) discuss the theoretical implications of aging, memory, and metacognition. It is important to note that the results found here might not generalize to JOLs, FOKs, or other types of metacognitive judgments. Finally, future research should establish the measurement and structural properties of metacognitive efficiency across age groups in order to more confidently assert that developmental similarities – or differences – obtain in metacognitive efficiency in the memory domain.

## Conclusion

Beyond age-equivalent metacognition, a major conclusion from the present study is that there is something special about associative metacognition. Both age groups showed greater metacognitive efficiency for associations compared to items. Additional internal cues and strategies related to binding word pairs might enhance the accuracy of their confidence rating during associative recognition. This builds on work demonstrating the power of cues, such as retrieval and/or response fluency, to support metacognition (e.g., Koriat, 2006; Thompson et al., 2013). However, additional work is needed to determine what mechanisms support enhanced associative metacognition in OAs and YAs. In the future, additional trial-level analyses, including neuroimaging techniques, could test whether underlying mechanisms that contribute to associative metacognition change as we age.

## Data Availability Statement

The raw data supporting the conclusions of this article will be made available by the authors, without undue reservation, to any qualified researcher.

## Ethics Statement

The studies involving human participants were reviewed and approved by University of Richmond Institutional Review Board. The participants provided their written informed consent to participate in this study.

## Author Contributions

AZ and JB were involved in the study conceptualization and experiment design, and analyzed the data. AZ and ES were involved in data collection. All authors contributed to the manuscript.

## Conflict of Interest

The authors declare that the research was conducted in the absence of any commercial or financial relationships that could be construed as a potential conflict of interest.

## Footnotes

1 See Supplementary Materials for the description and results of a pilot experiment with the same methods and procedures as the main study but different word stimuli.

2 https://tomfaulkenberry.shinyapps.io/anovaBFcalc/

3 OAs showed higher mean confidence than YAs (OA: *M* = 4.79, *SD* = 1.21; YA: *M* = 4.37, *SD* = 1.49) and this difference was significant, *t*(47) = 2.52, *p* < 0.05, Cohen’s *d* = 0.72. However, meta-*d’*, like *d’*, has been shown to be resistant to response bias, including metacognitive response bias (Maniscalco and Lau, 2012; Barrett et al., 2013).

4 See Supplementary Materials: Confidence Experiment, for a follow-up study to test effects of confidence on memory performance.
