# Supplementary material for: Evidence for Age-Equivalent and Task-Dissociative Metacognition in the Memory Domain
Source: Front Psychol. 2021 Feb 5;12:630143. doi: 10.3389/fpsyg.2021.630143 (PMC7901934; doi:10.3389/fpsyg.2021.630143)
Supplement: Supplementary file 1 [file Table_1.docx]

***Supplementary Material***

***1 Pilot Experiment***

A pilot experiment with different stimulus selection criteria but similar methods and procedure was conducted prior to the main study. The stimuli (standard nouns) in the pilot experiment (described below) were retrieved from the Medical Research Council (MRC) Psycholinguistic Database (Wilson, 1988) with word properties that matched those used by Palmer et al. (2014). See Supplementary Table 1 for stimulus characteristics in the pilot and the main study. The pilot experiment produced many high performers (13 out of 34 performed at 90% correct or better in the associative task; 8 out of 34 performed at 90% correct or better in the item task). For this reason, we expanded stimulus selection in the MRC database to include a larger range of words, creating more variability in the main study’s stimulus set. As a result, we made the current study more difficult. For example, in the main study, 4 out of 49 performed at 90% correct or better in the associative task; 2 out of 49 performed at 90% correct or better in the item task.

A second difference between the pilot and the main study is that older adults in the pilot were recruited exclusively from University of Richmond’s Osher Lifelong Learning Institute. These adults often participate in psychology experiments on campus and may have more experience with computerized tasks than the general older adult population. For this reason, in the main study, we excluded all Osher members as well as those who have participated in previous research in the Cognitive Aging Laboratory.

*1.1 Method*

*1.1.1 Design and Participants.* As in the main study, the pilot utilized a mixed design with age group (young, old) as a between-subjects factor and test type (item, associative) as a within-subjects factor. There were 35 participants, 26 older adults (19 female) aged 57-90 (*M* = 69.48, *SD* = 7.69) and 9 younger adults (3 female) aged 18-19 (*M* = 18.44, *SD* = 0.53). Younger adults were students recruited from introductory psychology classes and received course credit for participation. Older adults were recruited from University of Richmond’s Osher Lifelong Learning Institute. They received $20 for participation. The entire experiment lasted approximately one hour and twenty minutes. One participant (older adult) was dropped due to low performance (*d’* = 0.15 or below). The final dataset included 34 participants (25 OAs and 9 YAs). Demographic data and standardized scores on processing speed and vocabulary measures are reported in Supplementary Table 2.

*1.1.2 Materials.* The stimuli consisted of English words generated using the Medical Research Council Psycholinguistic Database (Wilson, 1988). See Supplementary Table 1 for stimulus properties. The same number of words as well as the same sorting and pairing methods were used as in the main experiment.

*1.1.3 Procedure.* Same as in the main study.

*1.1.4 Metacognitive efficiency.* Same as in the main study.

*1.2 Results and Discussion*

*1.2.1 Metacognitive Efficiency.* To test effects of age and test type on metacognition, we entered individuals’ meta-*d’/d’* scores into a two-way ANOVA with age group (YA, OA) as the between-participants factor and test type (item, associative) as the within-participants factor. There was a significant main effect of test type, *F*(1,32) = 18.75, *p* < .001, *η_p_^2^* = .370, but no significant main effect of age or interaction between age and test type (*F*s < 1.03). As in the main study, we found overall associative meta-*d’*/*d’* (*M* = 1.18, *SD* = 0.38) was significantly higher than item meta-*d’*/*d’* (*M* = .85, *SD* = .27), despite there being no significant difference in associative and item performance. The significant difference between item and associative metacognitive efficiency remained when younger adults (n = 9) were removed, *t*(24) = 3.15, *p* < .01, Cohen’s *d* = (associative *M* = 1.15, *SD* = 0.34; item *M* = .87, *SD* = 0.26).

*1.2.2 Recognition Memory Performance (d’).* We examined recognition memory performance scores (*d’*) in a two-way analysis of variance (ANOVA) with age group (YA, OA) as the between-participants factor and test type (item, associative) as the within-participants factor. No significant main effect of test type or interaction were found (*F*s < 2), replicating the main experiment’s results. There was also no main effect of age, *F* < 1. It is important to note a large difference in sample size between younger (n = 9) and older (n = 25) age groups, making any age effects and differences less likely in this dataset.

***1.3 Pilot Experiment Tables***

**Supplementary Table 1.** Stimulus Characteristics in the Pilot and Main Study.

|  | Pilot Experiment | | | Experiments 1 & 2 | | |
| --- | --- | --- | --- | --- | --- | --- |
|  | M (SD) | Min | Max | M (SD) | Min | Max |
| Number Letters | 5.37 (1.30) | 4 | 8 | 5.68 (1.43) | 4 | 10 |
| Number Syllables | 1.52 (0.64) | 1 | 3 | 1.55 (0.50) | 1 | 2 |
| Familiarity | 516.43 (53.89) | 400 | 700 | 539.2 (58.52) | 200 | 700 |
| Concreteness | 539.03 (67.29) | 400 | 700 | 462.52 (112.8) | 200 | 700 |
| Imageability | 543.24 (55.14) | 400 | 700 | 486.88 (96.4) | 200 | 700 |

*Note*. Standard nouns from the Medical Research Council (MRC) Psycholinguistic Database (Wilson, 1988) were used in all experiments.

**Supplementary Table 2.** *Pilot Experiment Means and Standard Deviations for Demographic Comparisons Between Age Groups*

| Variable | Younger adults (N = 9) | Older adults (N = 25) |
| --- | --- | --- |
| % Female | 33.3 | 72.0 |
| Age | 18.44 (0.53) | 68.63 (6.53) |
| Years of education | 12.61 (0.86) | 17.64 (2.22) |
| Self-rated health | 9.33 (0.71) | 8.60 (1.15) |
| Self-rated vision | 7.67 (3.04) | 8.20 (1.41) |
| Self-rated hearing | 9.11 (1.05) | 7.88 (2.03) |
| Speed of processing^a^ | 70.89 (16.50) | 47.68 (13.05) |
| Vocabulary^b^ | 24.67 (1.50) | 30.56 (2.98) |

*Note.* Scales for self-rated health, vision, and hearing ranged from 0 (poor) to 10 (excellent). a Digit Symbol Substitution Test (DSST; Wechsler, 1981). b Ekstrom, French, Harman, and Dermen (1976) Synonyms Test.

***2 Confidence Experiment***

We conducted a second experiment with younger adults in order to test possible effects of confidence ratings on memory performance in item and associative tasks. In the main experiment, the interaction between test type and age (with *d’* entered as the dependent variable) was not significant (*p* = .116). In the ADH literature, a significant Test Type x Age interaction is often cited to support the presence of an age-related associative deficit. However, the presence of confidence ratings in our memory tasks may have enhanced recognition performance, especially in the associative task (see Figure 3 in main text). Touron and Hertzog (2004) found that inserting post-decision confidence ratings in their noun-pair task increased use of memory retrieval strategies and enhanced item knowledge (also, see Double & Birney, 2017). They suggested that confidence ratings might have encouraged participants to reflect on associative recognition memory confidence, leading to retrieval strategy use. These findings as well as the main experiment’s performance data motivated us to test this effect of confidence judgments.

*2.1 Method*

*2.1.1 Design and Participants.* We utilized a two-factor design with confidence ratings (present, absent) and test type (item, associative) as within-subjects factors. There were 38 participants, (27 female) aged 18-22 (*M* = 19.47, *SD* = 1.16). All participants were students recruited from introductory psychology classes and received course credit for participation. The entire experiment lasted approximately one hour. Using the same exclusion criterion as in the main study, participants (7) were dropped due to low performance (*d’* = 0.15 or below). Three additional participants were dropped for not completing all experimental blocks. The final dataset included 28 participants. Demographic data and standardized scores on processing speed and vocabulary measures are reported in Supplementary Table 3.

*2.1.2 Materials.* Same as in the main study.

*2.1.3 Procedure.* Same as in the main study except for one major difference. During half of the experiment (2 out of 4 blocks), participants did not make confidence ratings during item and associative recognition tests. During testing in these no-confidence blocks, a new recognition trial began immediately following each ‘Y’ or ‘N’ response. The order of blocks with confidence ratings present (2) and absent (2) was counterbalanced across subjects. The order of task type (associative, item) was counterbalanced as well, just as in the main study.

*2.1.4 Metacognitive efficiency.* Same as in the main study.

*2.2 Results and Discussion*

*2.2.1 Metacognitive Efficiency.* Unlike the main experiment and pilot study (*Supplementary Material*), there was no significant difference between average item and associative meta-*d’*/*d’*, *t* < 2.

It is important to note that fewer trials were used to calculate meta*-d’/d’* in this follow-up study. Two out of four blocks collected confidence ratings, providing only 40 trials in the associative task and 80 trials in the item task for measurement of metacognition. For this reason, calibration may be less sensitive. Previous relevant studies used at least 100 trials with confidence ratings to calculate *meta-d’* (e.g., Palmer et al., 2014).

*2.2.2 Recognition Memory Performance (d’).* We entered performance (*d’*) into a two-way ANOVA with test type item, associative) and confidence block (ratings present, ratings absent) as within-participants factors. Here, we found a significant test type x confidence block interaction, *F*(1,27) = 10.404, *p* < .01, *η_p_^2^* = .278. Item performance was significantly lower when confidence ratings were present (*M* = 1.61, *SD* = .79) than when they were absent, (*M* = 1.96, *SD =* .84), *t*(27) = 2.57, *p* < .05, Cohen’s *d* = -0.43 (see Supplementary Figure 1). There was no significant difference in associative performance when confidence ratings were present (*M* = 1.80, *SD* = .99) compared to when they were absent (*M* = 1.70, *SD* = .95). This dissociation may have been driven by significantly higher false-alarm rates (FARs) in the item task with confidence ratings present (*M* = .20, *SD* = 0.15) compared to absent (*M* = .13, *SD* = 0.10), *t*(27) = 3.52, *p* = .002, Cohen’s *d* = 0.55. There was no significant difference between FARs in the confidence conditions for the associative task, *t* < 1. Also, hit rates (HR) were not significantly different between confidence conditions in either task, *t*s < 1. There was no main effect of test and no main effect of confidence block.

**2.3 Confidence Experiment Tables and Figures**

**Supplementary Table 3.** *Confidence Experiment Means and Standard Deviations for Demographic Information*

| Variable | N = 28 |
| --- | --- |
| % Female  Age  Years of education | 71.4  19.43 (1.23)  13.63 (1.12) |
| Self-rated health | 8.04 (1.45) |
| Self-rated vision | 9.14 (0.85) |
| Self-rated hearing | 8.89 (1.37) |
| Speed of processing^a^ | 66.04 (9.99) |
| Vocabulary^b^ | 25.41 (2.33) |

*Note.* Scales for self-rated health, vision, and hearing ranged from 0 (poor) to 10 (excellent). a Digit Symbol Substitution Test (DSST; Wechsler, 1981). b Ekstrom, French, Harman, and Dermen (1976) Synonyms Test.

*

***Supplementary Figure 1.*** Performance (*d’*) by task type (item, associative) and confidence block (ratings present, ratings absent). Errors bars show standard error.

***3 Gamma Coefficients***

The results from the metacognitive efficiency analyses provided compelling support for the age-equivalent monitoring hypothesis. To test this hypothesis further, we analyzed Goodman-Kruskal gamma coefficients, one of the most widely used measures in metacognitive aging (e.g., Eakin et al., 2014; Tauber & Dunlosky, 2012; Wong et al., 2012). A 2 (younger, older) X 2 (item, associative) ANOVA with gamma coefficients as the dependent variable supported the age-equivalence analyses of metacognitive efficiency. The main effect of age was nonsignificant, *F* (1,47) = 1.36, *p* = .249, *η_p_^2^* = .028). Mean gammas were equivalent between younger (*M =* .52, *SD* = .05) and older adults (*M =* .44, *SD* = .05). Moreover, these values were significantly greater than zero, both *t’*s > 9.00, both *p*’s < .001, indicating good metacognitive accuracy by both younger and older adults.

We further assessed the evidence favoring the null over the alternative hypothesis of the impact of age, this time, on gamma coefficients, by calculating Bayes factor (BF). The results yielded BF_01_ = 3.48, indicating that the observed data favored the null hypothesis of age equivalence for metacognitive accuracy over the alternative by about 3.5 to 1, M_dif_ = .08, *t*(47) = 1.17, *p* = .249. The posterior probability for the null is 0.7768 and for the alternative is 0.2232.

In contrast to the results for metacognitive efficiency, the main effect of test type on gamma coefficients was nonsignificant, *F* (1,47) = .243, *p* = .624, *η_p_^2^*= .005.

The age by test type interaction was nonsignificant, (*F* < 1).

**Supplementary References**

Double, K.S., & Birney, D.P. (2017). Are you sure about that? Eliciting confidence ratings may influence performance on Raven's progressive matrices. *Thinking and Reasoning, 23*(2), 190-206. doi: 10.1080/13546783.2017.1289121

Eakin, D. K., Hertzog, C., & Harris, W. (2014). Age invariance in semantic and episodic metamemory: Both younger and older adults provide accurate feeling-of-knowing for names of faces. Aging, Neuropsychology, and Cognition, 21(1), 27-51.

Ekstrom, R. B., French, J. W., Harman, H. H., and Dermen, D. (1976). Manual

for Kit of Factor- Referenced Cognitive Tests. Princeton, NJ: Educational Testing

Service.

Palmer, E. C., David, A. S., and Fleming, S. M. (2014). Effects of age on

metacognitive efficiency. Conscious. Cogn. 28, 151–160. doi: 10.1016/j.concog.

2014.06.007

Tauber, S. K., & Dunlosky, J. (2012). Can older adults accurately judge their learning of emotional information? Psychology and Aging, 27(4), 924-933.

Touron, D. R., & Hertzog, C. (2004). Distinguishing age differences in knowledge, strategy use, and confidence during strategic skill acquisition. *Psychology and*

*Aging, 19*(3), 452-466.

Wechsler, D. (1981). The Wechsler Adult Intelligence Scale - Revised. New York, NY:

Psychological Corporation.

Wilson, M. (1988). MRC psycholinguistic database: machine-usable dictionary,

version 2.00. Behav. Res. Methods Instrum. Comput. 20, 6–10. doi: 10.3758/

BF03202594

Wong, J. T., Cramer, S. J., and Gallo, D. J. (2012). Age-related reduction in the

confidence-accuracy relationship in episodic memory: effects of recollection

quality and retrieval monitoring. Psychol. Aging 27, 1053–1065. doi: 10.1037/

a0027686
